# A Low-Latency RDP-CORDIC Algorithm for Real-Time Signal Processing of Edge Computing Devices in Smart Grid Cyber-Physical Systems

**DOI:** 10.3390/s22197489

**Published:** 2022-10-02

**Authors:** Mingwei Qin, Tong Liu, Baolin Hou, Yongxiang Gao, Yuancheng Yao, Haifeng Sun

**Affiliations:** 1 School of Information Engineering, Southwest University of Science and Technology, Mianyang 621000, China; 2 Robot Technology Used for Special Environment Key Laboratory of Sichuan Province, Mianyang 621000, China; 3 School of Computer Science and Technology, Southwest University of Science and Technology, Mianyang 621000, China

**Keywords:** smart grid, edge computing, signal processing, CORDIC, scaling factor

## Abstract

Smart grids are being expanded in scale with the increasing complexity of the equipment. Edge computing is gradually replacing conventional cloud computing due to its low latency, low power consumption, and high reliability. The CORDIC algorithm has the characteristics of high-speed real-time processing and is very suitable for hardware accelerators in edge computing devices. The iterative calculation method of the CORDIC algorithm yet leads to problems such as complex structure and high consumption of hardware resource. In this paper, we propose an RDP-CORDIC algorithm which pre-computes all micro-rotation directions and transforms the conventional single-stage iterative structure into a three-stage and multi-stage combined iterative structure, thereby enabling it to solve the problems of the conventional CORDIC algorithm with many iterations and high consumption. An accuracy compensation algorithm for the direction prediction constant is also proposed to solve the problem of high ROM consumption in the high precision implementation of the RDP-CORDIC algorithm. The experimental results showed that the RDP-CORDIC algorithm had faster computation speed and lower resource consumption with higher guaranteed accuracy than other CORDIC algorithms. Therefore, the RDP-CORDIC algorithm proposed in this paper may effectively increase computation performance while reducing the power and resource consumption of edge computing devices in smart grid systems.

## 1. Introduction

**Figure 1:**
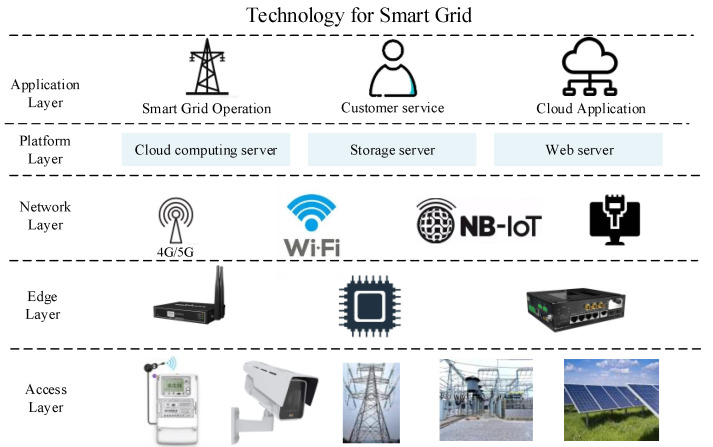
Basic Structure of Edge-enabled Smart Grids.

Over the past few years, cloud computing infrastructure has been the dominant solution used to handle heavy computational tasks related to smart grid applications [1]. With the rise of smart grid cyber–physical systems and the growing number of Internet of Things (IoT) devices, cloud computing can no longer satisfy all the computing needs of smart grid applications. Edge computing can move data processing tasks from remote cloud computing centers to devices at the edge of the network. Edge computing technology alleviates network congestion, latency and packet loss in smart grid architectures under cloud computing [2,3]. The architecture of edge-enabled smart grid cyber-physical systems is shown in Figure 1, which consists of an access layer, an edge layer, a network layer, a platform layer, and an application layer. Here, the ability to process intelligent edge data is provided by the edge computing devices at the Edge Layer.

Many scholars have proposed applications of edge computing, which are especially suitable for smart grids. A cloud edge collaborative intelligent method for object detection was proposed in the literature [4], and it is applied to insulator string recognition defect detection in the power IIoT. A fault detection method for pumping units based on edge intelligence, which effectively improves the fault detection accuracy while maintaining low computational requirements, was proposed in [5]. The important features of edge computing applied to smart grid mainly include: support for real-time [6,7,8,9,10] and low power [11,12,13,14,15] consumption. Data processing is the most time-consuming and energy-intensive part of edge computing. Furthermore, since edge computing devices cannot guarantee high-capacity storage, processing these large volumes of data is an important issue to be addressed [16].

**Figure 2:**
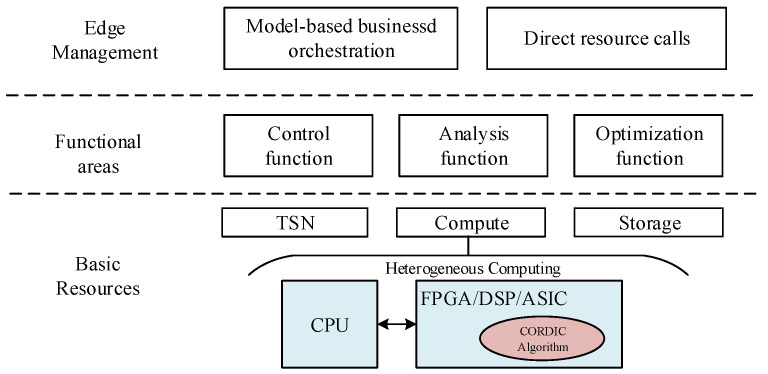
Edge computing framework based on heterogeneous computing.

Most of the current edge computing devices use an edge computing framework based on heterogeneous computing, as shown in Figure 2. In this framework, the computational power provided by edge devices mainly depends on hardware accelerators, which include Digital Signal Processing (DSP), Application Specific Integrated Circuit (ASIC) and Field Programmable Gate Array (FPGA). In the heterogeneous framework based on CPU+FPGA [17], FPGA has the characteristics of reconfiguration and energy efficiency. The literature [18] appropriately places DSP operators on edge devices, so that the edge layer can reduce the energy consumption of each event by as much as 4%. The edge computing device based on CPU+ASIC structure [19] has a significantly better acceleration ratio than the existing GPU+CPU method, and has the advantages of small size and low power consumption. A signal processing algorithm named CORDIC is often used in hardware to handle complex data computation problems in real-time. It can implement many complex functions and mathematical problems with simple addition, subtraction, and shift operations. Table 1 lists some applications of the CORDIC algorithm, including trigonometric functions [20], hyperbolic functions [21], FFT [22] and singular value decomposition [23]. Nonetheless, the computational speed of the conventional CORDIC algorithm is limited by the number of iterations, i.e., the more iterations of the CORDIC algorithm, the higher the computational accuracy and the longer the time delay. Therefore, reducing the number of iterations of the CORDIC algorithm, while ensuring the computational accuracy of the algorithm, can reduce the computational latency and hardware resource consumption.

In summary, this work aimed to discover a high-performance CORDIC algorithm. As a result, we proposed a RDP-CORDIC algorithm and implemented the hardware design of the algorithm. The RDP-CORDIC algorithm, characterized by fewer iterations, less hardware resource consumption and faster processing speed, can effectively improve the data processing speed and reduce the latency and power consumption of edge computing devices in smart grid cyber-physical systems. The main contributions of this work are as follows:

- We proposed a rotation direction prediction method of the CORDIC algorithm, which completed the calculation of all the micro-rotation directions by inputting the angle and direction prediction constants, providing the basis for the subsequent merge iteration;
- A constant compensation algorithm for direction prediction was proposed to achieve higher accuracy of direction prediction, being able to solve the problem of large memory consumption under the condition of high accuracy;
- The single-stage iterative structure of the CORDIC algorithm was replaced by a three-stage and multi-stage iterative structure. Based on this structure, the CORDIC algorithm design with high accuracy, low latency, and low power consumption was achieved.

## 2. Related Work

The CORDIC algorithm was proposed by Volder in 1959 and was later generalized by Walther. Subsequently, some other methods were proposed that aimed to enhance the precision and reduce iterations and resource consumption. Among them, Radix-4 CORDIC algorithms [24] worked on zero hopping technology to reduce the number of iterations for rotation to 50%. Later, a hybrid radix 2–4 CORDIC algorithm with high-performance compensation technique waspresented [25] with reduced number of iterations by 1/4, including scale factor calculation and compensation. Nevertheless, the computation and correction of variable scale factor was a focused issue for higher radix CORDIC algorithms [26,27,28,29] and advanced hybrid CORDIC algorithms [30]. The scale-free CORDIC algorithm [31,32] approximated the sine and cosine functions by the Taylor series, thereby eliminating the need for the scalar factors, except for a limited convergence range and poor accuracy. A new hybrid CORDIC algorithm was proposed [33] to be able to further reduce the latency of CORDIC by reducing the number of iterations equal to (3*N*/8) + 1. A technique reported in low latency CORDIC algorithm [34] utilized the binaryto-bipolar recoding (BBR) method to reduce the overall iterations to (*N* + 1)/3, with no scale factor compensation. Similar to [23], the CORDIC algorithms [35,36,37] cut down time and memory at the expense of accuracy. The CORDIC II algorithm proposed in [38] had excellent performance in terms of resource consumption and latency, but its low accuracy held it back. Table 2 lists some important features of the above related CORDIC algorithms, including the rotation radix, prediction of rotation direction and whether the scaling factor is fixed.

## 3. Conventional CORDIC Algorithm

**Figure 3:**
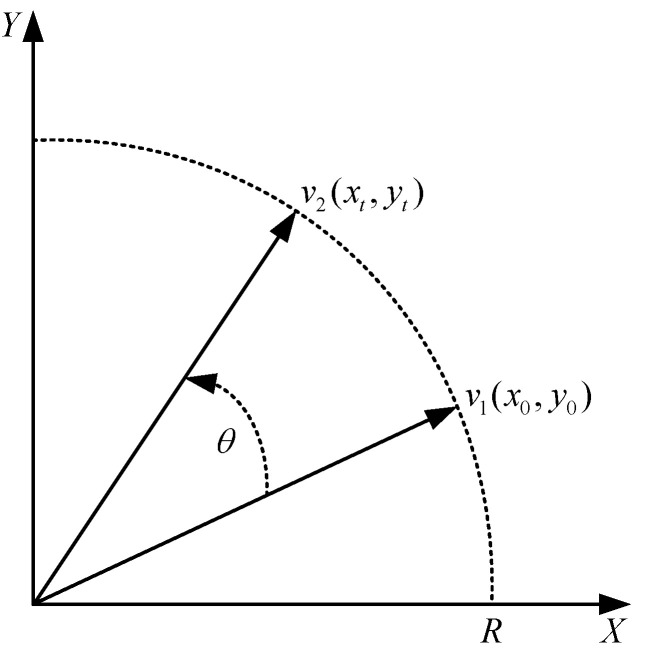
Rotation model for CORDIC algorithm.

The CORDIC algorithm contains two modes (rotation mode, vector mode) and three coordinate systems (circular coordinates, linear coordinates, hyperbolic coordinates). Different functions can be derived under different modes and different coordinate systems. The CORDIC algorithm rotation mode of the circle coordinate system was taken as an example to construct the simplest vector rotation model, as shown in Figure 3.

Suppose vector *v*_1_ is rotated by *θ* to obtain vector *v*_2_, and the coordinates of *v*_1_ and *v*_2_ are (*x*_0_, *y*_0_), (*x*_t_, *y*_t_) respectively, then, the equation of change of vector coordinates can be expressed by Equation (1).

(1) $$\left\{ \begin{array}{l} x_{t}=x_{0}\cos\theta -y_{0}\sin\theta =\cos\theta (x_{0}-y_{0}\tan\theta )\\ y_{t}=x_{0}\sin\theta +y_{0}\cos\theta =\cos\theta (y_{0}+x_{0}\tan\theta ) \end{array}\right.$$

By dividing the single rotation angle of Equation (1) into multiple directed rotations *θ_i_* = tan^−1^(2^−*i*^), each rotation can be expressed by the iterative Equation (2).

(2) $$\{\begin{array}{c} x_{i+1}=\cos\theta_{i}(x_{i}-d_{i}\cdot y_{i}\cdot 2^{-i})\\ y_{i+1}=\cos\theta_{i}(y_{i}+d_{i}\cdot x_{i}\cdot 2^{-i})\\ z_{i+1}=z_{i}-d_{i}\cdot \theta_{i} \end{array}\begin{array}{l} \begin{array}{cc} if(z_{i}>0) & d_{i}=1 \end{array}\\ \begin{array}{cc} else\;\;\; & d_{i}=-1 \end{array}\; \end{array}$$

where *z_i+_*_1_ indicates the remaining angle and *d_i_* is the direction of rotation. (*x_i+_*_1,_ *y_i_*_+1_) indicates the coordinates of (*x_i_*, *y_i_*) after the next rotation. In the rotation mode, the remaining angle *z_i_*_+1_ value was used as a direction reference, and after *n* iterations, the *z_i_*_+1_ value tended to zero and the vector *v_i_* almost tended to the vector, thus realizing the successive approximation calculation.

Since cos *θ_i_* in Equation (2) involved multiplication in the iterative calculation process, it can be proposed not to participate in the iterative operation. Let $K=\prod_{i=0}^{n}\cos\theta i\;$=1/[(1 + 2^−2*i*^)^0.5^], 1/*K* is the scaling factor mentioned above, then the iteration equations of the radix-2 CORDIC algorithm in rotation mode at the (*i* + 1)th step are as follows:

(3) $$\{\begin{array}{c} x_{i+1}=x_{i}-d_{i}\cdot y_{i}\cdot 2^{-i}\\ y_{i+1}=y_{i}+d_{i}\cdot x_{i}\cdot 2^{-i}\\ z_{i+1}=z_{i}-d_{i}\cdot \tan^{-1}(2^{-i}) \end{array}\begin{array}{l} \begin{array}{cc} if(z_{i}>0) & d_{i}=1 \end{array}\\ \begin{array}{cc} else\;\;\; & d_{i}=-1 \end{array}\; \end{array}$$

To facilitate the subsequent verification of the performance of the CORDIC algorithm, the principles for computing the sine and cosine functions are described below. Combining Equation (1) with Equation (3), a formula for the CORDIC algorithm that calculates the sine and cosine functions can be introduced. If given *z*_0_ = θ, the coordinates of Equation (3) are (*x_n_*, *y*_*n*_) after *n* iterations of calculation.

(4) $$\left(\begin{array}{c} x_{n}\\ y_{n} \end{array}\right)\approx \frac{1}{K}\cdot \left(\begin{array}{c} x_{0}\cos\theta -y_{0}\sin\theta \\ x_{0}\sin\theta +y_{0}\cos\theta \end{array}\right)$$

From Equation (4), it can be found that taking *x*_0_ = *K* and *y*_0_ = 0, after *n* iterations of calculation, *x_n_* and *y_n_* will be equal to the values of cos *θ* and sin *θ*, respectively. Therefore, the calculation of sine and cosine functions based on the CORDIC algorithm was implemented.

## 4. RDP-CORDIC Algorithm

The micro-rotation direction of the conventional CORDIC algorithm is determined by the remaining angle after the last iteration, which leads to the problem of high latency. Although the high latency problem may be solved by way of a parallel pipeline structure, it increases the hardware resource overhead, and the most effective way to solve the high latency is to reduce the number of iterations. In this paper, a rotation direction prediction CORDIC (RDP-CORDIC) algorithm was proposed to reduce the number of iterations by calculating all micro-rotation directions in advance, so that the conventional single-stage iterative structure could be changed into a multi-stage iterative structure. The current direction prediction algorithms mainly include the Booth encoding method and the binary-to-bipolar recoding (BBR) method. The Booth encoding method is responsible for predicting the direction of rotation after [*N* − log_2_^3^]/3 iterations, thus reducing the number of iterations by about 1/2. The BBR is impressed by decomposing the input angle *θ* into a combination of a larger angle and several 2^−*i*^ radians so that the direction of rotation is determined by the binary bit value of *θ* each rotation. Note that, the BBR method requires a ROM to store all the computation results after *N*/3 − 1 iterations, and the ROM consumption increases as precision gets higher, e.g., 16-bit precision requires a ROM of 26 × 16 × 2 (bit) size.

### 4.1. Rotation Direction Prediction

Considering that the BBR method allows the binary bit value of angle to represent the direction of micro-rotation, i.e., $\theta =\sum_{i=0}^{\infty }d_{i}2^{-i}={\left(d_{\theta }\right)}_{2}$, this method fixes the rotation angle as 2^−*i*^, resulting in large consumption of ROM resources. Therefore, the micro-rotation angle chosen for the RDP-CORDIC algorithm was tan^−1^(2^−*i*^), and a new rotation direction prediction method needs to be sought.

The input angle θ∈[0,π/4] can be expressed as:

(5) $$\theta =\overset{n=\infty }{\underset{i=0}{\sum }}\sigma_{i}\tan^{-1}\left(2^{-i}\right)$$

where,$\;\sigma_{i}\in \left\{-1,1\right\}$, *i* ≥ 1. Let $\sigma_{i}=2d_{i}-1$, $d_{i}\in \left\{0,1\right\}$, at which point *s* = 2, then $\theta =\sum_{i=1}^{n=\infty }\left(2d_{i}-1\right)\tan^{-1}\left(2^{-i}\right)$, and Equation (6) could be derived.

(6) $$\begin{array}{l} \theta =\sum_{i=1}^{\infty }\left(2d_{i}-1)\tan^{-1}(2^{-i}\right)\\ =\sum_{i=1}^{\infty }\left(2d_{i}-1\right)(2^{-i}-\frac{2^{-3i}}{3}+\frac{2^{-5i}}{5}-\frac{2^{-7i}}{7}+\cdots )\\ =\sum_{i=1}^{\infty }\left(2d_{i}-1\right)(2^{-i})-\sum_{i=1}^{\infty }\left(2d_{i}-1\right)(\frac{2^{-3i}}{3}-\frac{2^{-5i}}{5}+\frac{2^{-7i}}{7}-\cdots )\\ =2d_{\theta }-1+\sum_{i=1}^{\infty }\left(2^{-i}-\tan^{-1}(2^{-i})\right)-2\sum_{i=1}^{\infty }d_{i}(2^{-i}-\tan^{-1}(2^{-i})) \end{array}$$

Let $\varepsilon =\sum_{i=1}^{\infty }\left(2^{-i}-\tan^{-1}\left(2^{-i}\right)\right)$, $\lambda =\sum_{i=1}^{\infty }d_{i}(2^{-i}$−$\tan^{-1}$($2^{-i}$)), it came from Taylor Formula that when $i\geq \left[\left(N-\log_{2}3\right)/3\right]$, $2^{-i}≅\tan^{-1}\left(2^{-i}\right)$, *λ* could be reduced to Equation (7), where [*] means to take an integer greater than or equal to *.

(7) $$\lambda =\sum_{i=1}^{\left[(N-\log_{2}3)/3\right]}d_{i}(2^{-i}-\tan^{-1}(2^{-i}))$$

*ε* is a constant of about 0.0421115429, the final rotation direction prediction formula is introduced as in Equation (8).

(8) $$\begin{array}{l} d_{\theta }=0.5\theta +0.5-0.5\varepsilon +\lambda \\ \;=0.5\theta +\lambda +0.478944228537446 \end{array}$$

From Equation (8), the direction of rotation could be calculated by entering the angle and *λ*. The binary bit value of the final calculation pointed the direction of rotation. Equation (6) gave the calculation of the value of *d*_1_, *d*_2_, *d*_3_, *d*_4_ and *d*_5_ in various combinations for 16-bit precision. In order to determine the rules for the value of *λ*, the cumulative value of the rotation angle corresponding to *λ* was viewed as the angle reference, which was denoted as *θ*_cp_. It should be noted that in the calculation, the values for *d*_6_~*d*_16_ were 0. When calculating *θ*_cp_, not only the sum of the angles of *d*_1_~*d*_5_ rotation may be covered, but also the micro-selected rotation angles of *d*_6_~*d*_16_ should be accumulated. Equation (9) is the calculation of the reference angle value *θ*_cp(*m*)_.

(9) $$\theta_{\mathrm{cp}(m)}=\sum_{i=1}^{m}\left(2d_{i}-1)\tan^{-1}(2^{-i}\right)-\sum_{i=m+1}^{16}\tan^{-1}(2^{-i})$$

Looking at the interval range of the input angle size, the redundant data is removed and the final direction prediction constants are shown in Table 3. The final direction calculation result is affected by the accuracy of the direction prediction constants *λ* and *θ*. In order to satisfy the accuracy of *d_θ_*, it is necessary to make the accuracy of *λ* higher than that of *d_θ_*. For the 16-bit precision of *d_θ_*, λ needs 17-bit size precision. Since the integer bits of both λ and *θ* are 0, each prediction constant in ROM only needs 16 bits in size. Therefore, to implement the RDP-CORDIC algorithm with *N* bit precision, the size of the prediction constants λ and *θ* in ROM is also the same as *N* bit.

The minimum angular reference value *λ*_cp__5_ for different values of *λ* is given in Table 3. The process of rotation direction prediction can be summarized as follows:

1. Compare the input angle with *θ*_cp_ in the direction prediction constant, and select the value of *λ* corresponding to a value close to and less than or equal to *θ*_cp_;
2. The binary value *d_θ_* representing the micro-rotation direction was calculated based on λ. Finally, the prediction of the micro-rotation direction in the non-iterative case was performed.

### 4.2. ROM Resource Optimization

A 14 × 16 × 2 bit ROM resource was required to make the above-mentioned 16-bit precision direction prediction. According to the theory of rotation direction prediction algorithm proposed in Section 4.1, the *N* bit width accuracy required a ROM of 2^[(*N*−log^_2_^3)/3]^ × *N* bit size, and the ROM consumption increased sharply with the increase of accuracy. The reason for the sharp increase of ROM consumption was that the high accuracy direction prediction asked for more *λ* values to be selected, leading to an increase in the table of direction prediction constants. It may be useful to analyze the *λ* expansion and let *m* = [(*N* − log_2_^3^)/3)], then *λ_m_* and *λ_m+_*_1_ are as in Equations (10) and (11), respectively.

(10) $$\lambda_{m}=\sum_{i=1}^{m}d_{i}(2^{-i}-\tan^{-1}(2^{-i}))$$

(11) $$\begin{array}{l} \lambda_{m+1}=\sum_{i=1}^{m+1}d_{i}(2^{-i}-\tan^{-1}(2^{-i}))\\ \;\;=\lambda_{m}+d_{m+1}(2^{-m-1}-\tan^{-1}(2^{-m-1})) \end{array}$$

Let *μ_i_* = 2^−*i*^ − tan^−1^(2^−*i*^), and the first 10 iterations of *μ_i_* are given in Table 4.

Combined with Taylor’s formula, when *m* ≥ [(*N* + log_2_(3/20) − 3)/5], Equation (11) can be reduced to Equation (12)

(12) $$\lambda_{m+1}=\lambda_{m}+d_{m+1}\cdot 2^{-3}\cdot \mu_{m}$$

Equation (12) is the relationship between *λ_m_*_+1_ and *λ_m_*, and similarly, the value of *λ_m_*_+*i*_ can be calculated from *λ_m_*. Thus, an accuracy compensation algorithm for *λ* is proposed, where *λ* is composed of a fixed *λ_s_* and an accuracy compensation *λ_c_*.

(13) $$\lambda =\lambda_{s}+\lambda_{c}$$

In Equation (13), *s* = [(*N* + log_2_(3/20) − 3)/5], the accuracy compensation *λ_c_* is calculated as Equation (14).

(14) $$\lambda_{c}=\sum_{i=s+1}^{m}d_{i}\cdot \mu_{s+1}\cdot 2^{-3(i-s-1)}$$

Since the accuracy of the rotation direction that can be derived is equal to *s* × 3 + log_2_3 > *m*, *d_i_* in Equation (14) can be calculated based on *λ_s_*. To sum up, the ROM consumption of the direction prediction constant was reduced from $2^{\left[N-\log_{2}3\right]/3}*N\;$ bit to $2^{\left[N+\log_{2}\left(3/20\right)-3\right]/5}*N$ bit by using the direction prediction constant accuracy compensation method, which achieved high accuracy and reduced the ROM consumption.

### 4.3. Iterative Merging

After getting the direction of rotation, the conventional single-stage iterative calculation Equation (3) now can be changed to a three-stage combined iteration, as shown in Equation (15).

(15) $$\left\{ \begin{array}{l} x_{i+3}=x_{i}(1-\frac{d_{i}\cdot d_{i+1}}{2^{\left(2i+1\right)}}-\frac{d_{i}\cdot d_{i+2}}{2^{\left(2i+2\right)}}-\frac{d_{i+1}\cdot d_{i+2}}{2^{\left(2i+3\right)}})-y_{i}\cdot \sum_{j=i}^{i+2}\left(2^{-j}\cdot d_{j}\right)+y_{i}\cdot \frac{d_{i}\cdot d_{i+1}\cdot d_{i+2}}{2^{\left(3i+3\right)}}\\ y_{i+3}=y_{i}(1-\frac{d_{i}\cdot d_{i+1}}{2^{\left(2i+1\right)}}-\frac{d_{i}\cdot d_{i+2}}{2^{\left(2i+2\right)}}-\frac{d_{i+1}\cdot d_{i+2}}{2^{\left(2i+3\right)}})+x_{i}\cdot \sum_{j=i}^{i+2}\left(2^{-j}\cdot d_{j}\right)-x_{i}\cdot \frac{d_{i}\cdot d_{i+1}\cdot d_{i+2}}{2^{\left(3i+3\right)}} \end{array}\right.$$

When the number of iterations *i >* [(*N* − 3)/3], *x_i_* 2^−(3*i*+3)^ and *y_i_* 2^−(3*i*+3)^ dropped to machine zero, Equation (15) removed $y_{i}\cdot \frac{d_{i}\cdot d_{i+1}\cdot d_{i+2}}{2^{\left(3i+3\right)}}$ and $x_{i}\cdot \frac{d_{i}\cdot d_{i+1}\cdot d_{i+2}}{2^{\left(3i+3\right)}}$ two terms, the three-level combined iteration formula became Equation (16).

(16) $$\left\{ \begin{array}{l} x_{i+3}=x_{i}(1-\frac{d_{i}\cdot d_{i+1}}{2^{\left(2i+1\right)}}-\frac{d_{i}\cdot d_{i+2}}{2^{\left(2i+2\right)}}-\frac{d_{i+1}\cdot d_{i+2}}{2^{\left(2i+3\right)}})-y_{i}\cdot \sum_{j=i}^{i+2}\left(2^{-j}\cdot d_{j}\right)\\ y_{i+3}=y_{i}(1-\frac{d_{i}\cdot d_{i+1}}{2^{\left(2i+1\right)}}-\frac{d_{i}\cdot d_{i+2}}{2^{\left(2i+2\right)}}-\frac{d_{i+1}\cdot d_{i+2}}{2^{\left(2i+3\right)}})+x_{i}\cdot \sum_{j=i}^{i+2}\left(2^{-j}\cdot d_{j}\right) \end{array}\right.$$

When *i >* [(*N* − 1)/2], *x_i_* 2^−(2*i*+1)^ and *y_i_* 2^−(2*i*+1)^ are machine zeros, and the subsequent iterative process can be represented by the multilevel merge iteration of Equation (17).

(17) $$\left\{ \begin{array}{l} x_{i+n}=x_{i}-y_{i}\cdot \sum_{j=i}^{i+n}2^{-j}\cdot d_{j}\\ y_{i+n}=y_{i}+x_{j}\cdot \sum_{j=i}^{i+n}2^{-j}\cdot d_{j} \end{array}\right.$$

In summary, the flow of rotation iteration is as follows:

1. For the number of iterations *i* ≤ [(*N* − 3)/3], the three-stage merge iteration Formula (15) was used;
2. When [(*N* − 3)/3] < *i* ≤ [(*N* − 1)/2], the three-stage merge iteration simplified Formula (16) was used;
3. Finally when *i* > [(*N* − 1)/2], Formula (16) for multi-stage merge iteration calculation was used.

## 5. Hardware Design of RDP-CORDIC Algorithm

The main hardware structures for implementing the CORDIC algorithm are loop iterative structures and pipelined iterative structures. The loop iterative structure is simple in design and consumes less hardware, but the computation speed slows down as the accuracy increases. The pipelined iterative structure is more complex and consumes more hardware, but the computation speed is much higher. For edge computing devices used in smart grid cyber-physical systems, the faster pipelined iterative structure is more suitable.

### 5.1. RDP-CORDIC Algorithm Structure Design

**Figure 4:**
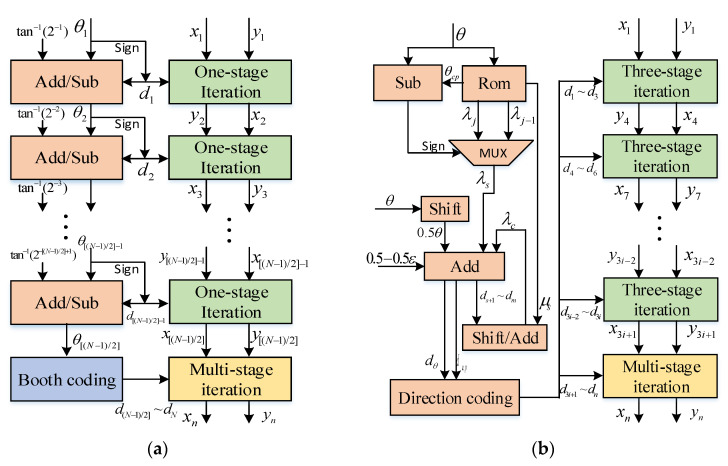
Structure of the classical optimized CORDIC algorithm and the RDP_CORDIC algorithm: (**a**) Classical optimized CORDIC algorithm pipeline structure; (**b**) Structure of RDP-CORDIC algorithm.

The pipeline structures of the classical optimized CORDIC algorithm and the RDP-CORDIC algorithm are shown in (a) and (b) of Figure 4, respectively. The direction of each rotation of the classical optimized CORDIC algorithm depends on the sign of the remaining angle *θ_n_*, and the result needs to be iteratively calculated in a [(*N* − 1)/2] stage pipeline method. The structure of RDP-CORDIC algorithm consists of two parts: the direction prediction part on the left side and the rotation iteration on the right side. The direction prediction module calculates all micro-rotation directions in advance, while the rotation iteration part transforms the single-stage iterative structure into a three-stage and multi-stage iterative structure since all micro-rotation directions are known. The new structure cut off part of hardware overhead and latency compared to the conventional structure.

As shown below, the workflow of the RDP-CORDIC algorithm consists of three steps (Algorithm 1).

**Algorithm 1** RDP-CORDIC workflow1. Directional rough prediction (1) Pre-store the direction prediction constants *θ*_cp_, *λ*_s_, and *μ_i_* in a ROM of size 2[(*N* − (log2(3/20) − 3)/5] bits; (2) Use the MSB of the input angle *θ* as the lookup address of the ROM for reading out *θ*_cp_; (3) Send the sign bit of the value of the input angle *θ* minus *θ*_cp_ to the selection input port of the multiplexer, and the multiplexer outputs the corresponding value of *λ*_s_; (4) Add up *λ*_*s*_, *θ*, and 0.5 − 0.5*ε* to get the rough rotation direction prediction value *d*_ap_.2. Accurate direction prediction (1) Shift and sum up the rough rotation direction prediction *d*_*s*+1_~*d_m_* with μs according to Equation (14) to calculate the compensation value *λ*_*c*_. (2) Calculate the exact direction prediction value *d*_*θ*_ by re-summing *λ*, *θ* and 0.5 − 0.5*ε*. 3. Iteration calculation (1) In the iterative calculation part uses multiple three-level merge iteration modules and one multi-level merge iteration module; (2) Set the input values of the iterative calculation module as *x*_1_ = *K* and *y*_1_ = 0; (3) The rotation directions *d*_1_~*d*_3*s*−1_ are determined by the rough direction value dap, and the rotation directions *d*_3*s*_~*d_n_* are determined by the accurate direction value *d*_*θ*_.

### 5.2. Calculation of Sine and Cosine Function Based on RDP-CORDIC Algorithm

To verify the performance of the RDP-CORDIC algorithm, the RDP-CORDIC algorithm was arranged to calculate the sine and cosine functions. Since the selected initial rotation angle value was tan^−1^(2^−*i*^), where *i* was an integer greater than 0, the calculated angle range was limited to [0, π/4]. The symmetry of trigonometric function were combined with the trigonometric change to expand the input angle range, and the final change relationship is shown in Table 5.

**Figure 5:**
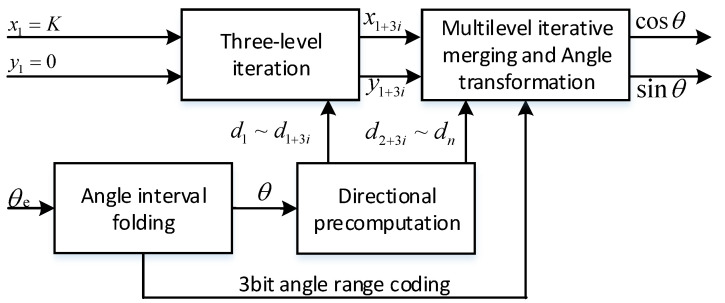
Implementation of RDP-CORDIC algorithm with sine and cosine functions.

**Figure 6:**
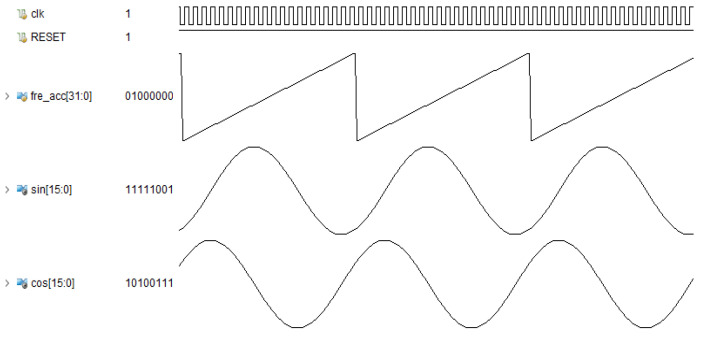
Simulation waveforms of calculated sine and cosine functions.

Based on the RDP-CORDIC algorithm, the implementation architecture of sine and cosine function calculation is shown in Figure 5, including an angle interval folding module, a direction prediction module, multiple three-stage iteration modules, a multi-stage merge iteration, and a triangular constant change module. The working principle is that the angle interval folding module transforms the input angle of any size into the interval [0~2π] and then sends the 3 bit angle range code to the angle transformation module.The rotation direction prediction module calculates all micro-rotation directions in advance based on the input angle, and then passes the direction values to the back-end iterative calculation module; after three-stage of running iterations and multi-stage of combined iterations, the calculated sine and cosine function values are output. Finally, the sine and cosine signals obtained by simulation with Vivado’s Simulation software are shown in Figure 6.

### 5.3. More Applications of the RDP-CORDIC Algorithm

As described in the introduction section, the RDP-CORDIC algorithm can also be used in more areas of smart grids. In a smart grid system, the frequency of the power system is an important indicator of power quality and needs to be detected in real time. If there is a problem in a section of the smart grid, the source of the fault can be cut off in time to protect the grid. Based on CORDIC algorithm to implement FFT, it can efficiently measure the higher harmonic and interference noise of power signal. The core of FFT implementation using CORDIC algorithm is to use CORDIC algorithm to implement complex multiplication operations in FFT. The complex multiplication operation in FFT is as in Equation (18).

(18) $$X_{k}=X_{0}*W_{N}^{nk}$$

where *X*_0_ = *x*_0_ + *j* × *y*_0_, *X_k_* = *x_k_* + *j* × *y_k_*, bringing $\;W_{N}^{nk}=e^{-j\frac{2\pi }{N}nk}$ into Equation (18), the imaginary and real parts of *X_k_* after being simplified are as in Equation (19).

(19) $$\left\{ \begin{array}{l} x_{k}=x_{0}\cos(-\frac{2nk\pi }{N})-y_{0}\sin(-\frac{2nk\pi }{N})\\ y_{k}=y_{0}\cos(-\frac{2nk\pi }{N})+x_{0}\sin(-\frac{2nk\pi }{N}) \end{array}\right.$$

The multiplication of the complex sequence and the rotation factor can be seen as the vector *X*_0_ rotated by *θ* = −2*nk*π/*N*, Then, using the CORDIC algorithm idea, we can transform Equation (19) into Equation (1). So the complex multiplication of FFT can then be implemented by the RDP-CORDIC algorithm.

In addition, the CORDIC algorithm can also realize singular value decomposition (SVD) for image denoising, data compression, etc. With the increase of matrix dimension, the computation volume of SVD grows exponentially, which has a great impact on the computation real-time of edge computing devices. Taking the 2 × 2 matrix G as an example, its bilateral Jacobi SVD algorithm was calculated as Equations (20) and (21).

(20) $$\left[\begin{array}{cc} \cos\theta_{L} & -\sin\theta_{L}\\ \sin\theta_{L} & \cos\theta_{L} \end{array}\right]\left[\begin{array}{cc} a & b\\ c & d \end{array}\right]\left[\begin{array}{cc} \cos\theta_{R} & -\sin\theta_{R}\\ \sin\theta_{R} & \cos\theta_{R} \end{array}\right]=\left[\begin{array}{cc} \delta_{1} & 0\\ 0 & \delta_{2} \end{array}\right]$$

(21) $$\left\{ \begin{array}{l} \theta_{R}+\theta_{L}=\arctan(\frac{c+b}{d-a})\\ \theta_{R}-\theta_{L}=\arctan(\frac{c-b}{d+a}) \end{array}\right.$$

**Figure 7:**
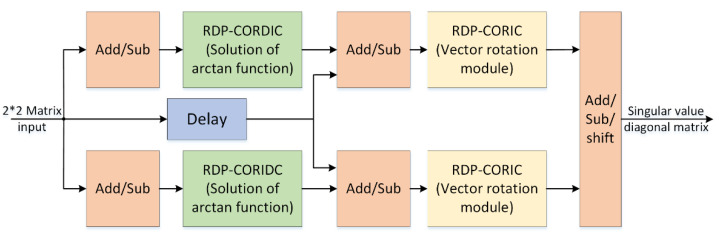
Structure of 2 × 2 SVD module based on RDP-CORDIC algorithm.

In Equation (20), *a*, *b*, *c*, *d* are the four elements of the second-order matrix *G*. *θ*_*R*_ and *θ*_*L*_ are the left and right rotation angles, calculated by Equation (21). The values of $\delta_{1}$ and $\delta_{2}$ are the singular values of the matrix *G*. In the above operation, both the arc tangent funcion and the sine/cosine function can be implemented by the CORDIC algorithm. The structure of the 2 × 2 SVD module based on the RDP-CORDIC algorithm is shown in Figure 7.

## 6. Performance Testing and Analysis

The hardware design of the 16-bit fixed-point decimal RDP-CORDIC algorithm was implemented on the Xilinx Kintex7 325T series FPGA hardware platform using Verilog HDL. In the first place, the effect of ROM resource optimization of the predictive direction CORDIC algorithm proposed in Section 4.2 was tested, and the size of RAM resources consumed before and after the optimization was compared. Subsequently, the proposed RDP-CORDIC algorithm was tested and compared with other related CORDIC algorithms in terms of latency, resource consumption, and power consumption. Based on the hardware structure of the RDP-CORDIC algorithm, the maximum absolute value errors of the sine and cosine functions, logarithmic function, square root function, hyperbolic sine, and hyperbolic cosine function were also tested at 16-bit accuracy. Finally, we analyze the time and maximum absolute value errors of the sine and cosine functions computed using the RDP-CORDIC algorithm.

### 6.1. ROM Optimization Results of the RDP-CORDIC Algorithm

**Figure 8:**
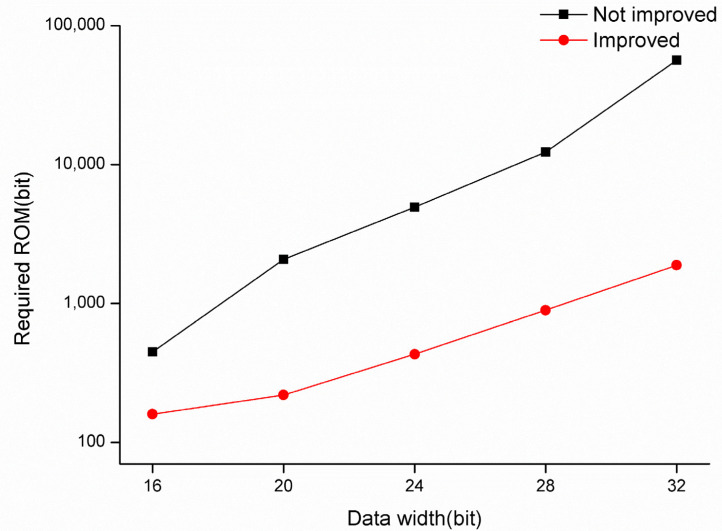
ROM resources required at different bit widths before and after optimization.

Figure 8 shows the comparison of ROM resource consumption before and after ROM optimization for this algorithm, from which it can be seen that the ROM consumption before the optimization is much higher than that after optimization. For 16-bit precision, the unoptimized algorithm requires a ROM size of 448 bits., while the optimized algorithm requires only 160 bits. for 32-bit precision, the unoptimized algorithm requires a ROM size of 56,320 bits, while the optimized algorithm consumes only 1888 bits. as the data bit width increases, the difference in ROM consumption before and after optimization increases significantly.

### 6.2. Performance Comparison of CORDIC Algorithms

The test results of the RDP-CORDIC algorithm and other related CORDIC algorithms in terms of latency and resource consumption are shown in Table 6. Apparently, in terms of latency, the R-4 CORDIC, R-8 CORDIC and Mixed-R CORDIC reduced latency but increased ROM consumption and had high hardware complexity. The RDP-CORDIC algorithm had a 70% lower latency compared to the conventional CORDIC algorithm, being parallel to the BBR-CORDIC algorithm. In terms of resource consumption, the RDP-CORDIC algorithm was similar to the CORDIC II algorithm, but the CORDIC II algorithm displayed a larger latency. Although the ROM consumption of the new algorithm was slightly higher than that of the conventional R-2 CORDIC algorithm, the RDP-CORDIC algorithm was clearly more advantageous, exchanging the ultra-small ROM capacity for 70% latency and 40% other resource consumption. In terms of power consumption, the proposed RDP-CORDIC algorithm facilitated an ultra-low power consumption of 28 mW. A comprehensive comparison showed that the RDP-CORDIC algorithm illustrated some advantages over other CORDIC algorithms in terms of latency, resource consumption and power consumption.

### 6.3. Test of Calculation Error and Calculation Time of Variousfunctions

**Figure 9:**
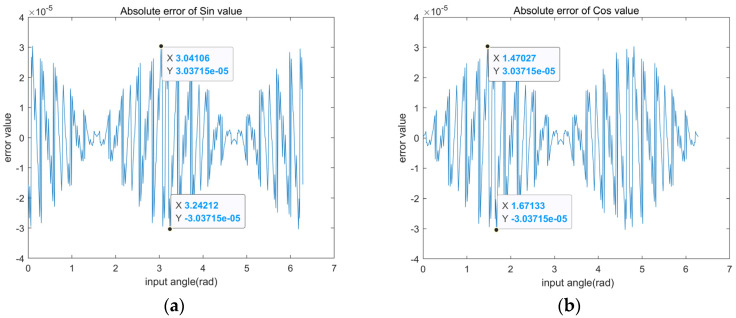

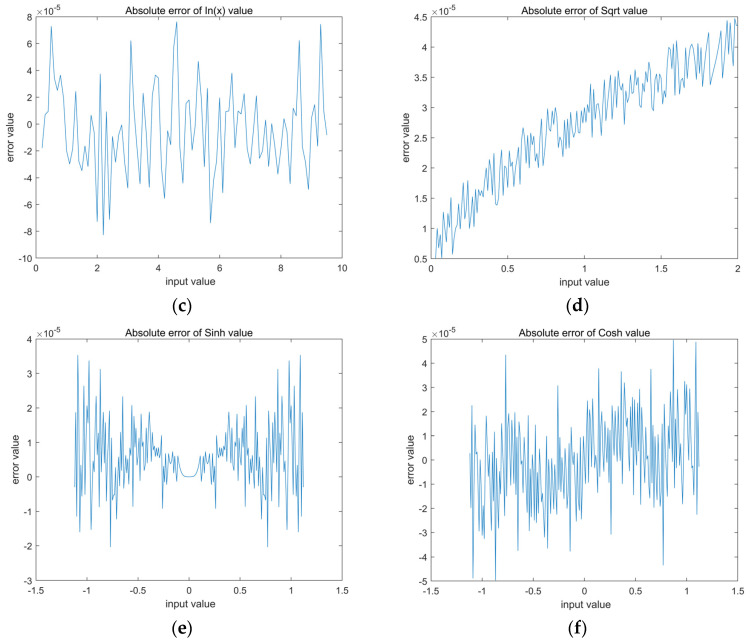
The various functions calculation error base on RDP-CORDIC algorithm: (**a**) Absolute error of sine signal value; (**b**) Absolute error of cosine signal value; (**c**) Absolute error of ln(*x*) value; (**d**) Absolute error of sqrt value; (**e**) Absolute error of sinh value; (**f**) Absolute error of cosh value.

Figure 9 shows the absolute error curves for the calculation of sine and cosine functions, logarithmic function, sqrt function and hyperbolic sine and hyperbolic cosine functions based on the RDP-CORDIC algorithm at 16-bit data width. The maximum magnitude error of the sine and cosine functions is clearly less than 3.04 × 10^−5^. The reason for the different errors for each input angle is that the error is 0 only when the accumulated value of the angle of directional rotation is equal to the input angle. However, the direction of rotation is not certain for different input angles, which results in the difference between the totalized rotation value and the input angle value. For other functions, the input test data is limited to a different range due to the characteristic limitations of the CORDIC algorithm. As in Figure 9c–f, the input angles are limited to [0.2, 9.5], [0.03, 2], [−1.12, 1.12] and [−1.12, 1.12], respectively. The test results show that the RDP-CORDIC algorithm performs well on a variety of functions, with maximum absolute errors less than 7.7 × 10^−4^. Because the computation time of each function is the same through the CORDIC algorithm, the sine and cosine functions are used for the test computation time. The time of the single computation of the sine and cosinefunctions for different CORDIC algorithms are compared in Table 7, and it can be found that the RDP-CORDIC algorithm takes only 60 ns at a system clock of 100 MHz.

**Figure 10:**
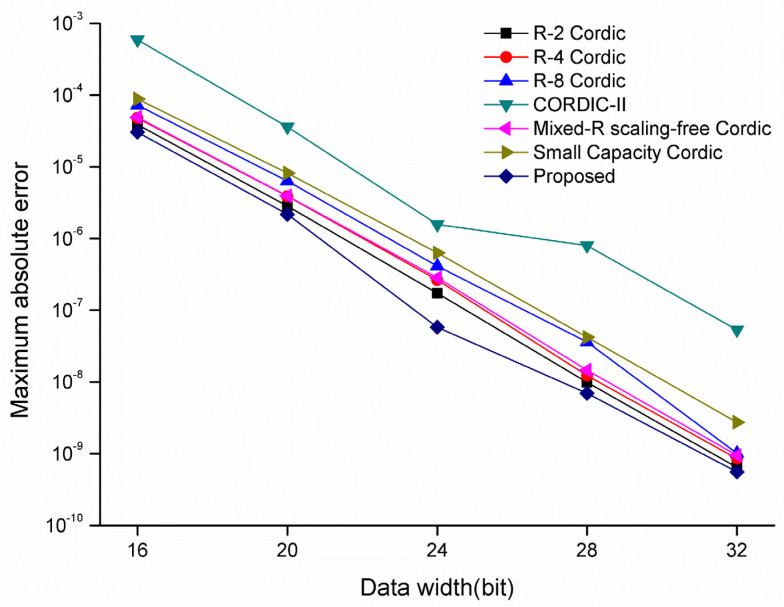
Maximum absolute error of sine and cosine signals calculated by various CORDIC algorithms at different bit widths.

Finally, the maximum absolute error of sine and cosine functions realized with each algorithm under different bit widths was tested, and the test results are shown in Figure 10. The results show that the RDP-CORDIC algorithm maintains the optimal performance in various bit width cases, and the error of the RDP-CORDIC algorithm is much lower than that of the CORDIC II algorithm, with similar resource consumption to that of the RDP-CORDIC algorithm. The reason why the RDP-CORDIC algorithm has higher accuracy compared with other algorithms is that the RDP-CORDIC algorithm calculates the rotation direction by formula once before iteration, while other CORDIC algorithms need to calculate the rotation direction each time, and multiple calculations may cause accuracy degradation.

## 7. Conclusions

Edge computing devices used in smart grid cyber-physical systems require real-time high-speed data processing capabilities with low power requirements. The CORDIC algorithm is widely used as a high-speed real-time numerical computation algorithm in hardware accelerator for edge computing devices. Limited by the excessive number of iterations and resource consumption, conventional CORDIC algorithms are too large to perform well in edge computing of smart grids. In this paper, a RDP-CORDIC algorithm was proposed in attempt to predict all micro-rotation directions in the non-iterative case, and then transform the conventional single-stage iteration structure into three-stage combined iteration and multi-stage combined iteration structure. An accuracy compensation algorithm for the direction prediction constants of the RDP-CORDIC algorithm was also proposed, which reduces the ROM consumption to 0.33% of the original at 16-bit accuracy. Finally, the hardware design of RDP-CORDIC algorithm was implemented on Xilinx Kintex7 325T series FPGA platform, along with the calculation of sine function, cosine functions, logarithmic function and other functions, accordingly. The test results showed that the RDP-CORDIC algorithm was plainly superior to other CORDIC algorithms in terms of latency, resource consumption and power consumption. The time and the maximum absolute error of the sine and cosine functions computed by the RDP-CORDIC algorithm also had some advantages over other CORDIC algorithms. It was experimentally confirmed that the proposed RDP-CORDIC algorithm was able to reduce resource consumption and increase the computational speed while maintaining the computational accuracy compared to other improved CORDIC algorithms. In the edge computing for signal processing of smart grid cyber-physical systems, the RDP-CORDIC algorithm exhibited its potential to effectively improve the speed of real-time data processing and reduce the power consumption of edge computing. The application of the RDP-CORDIC algorithm will be the focus of our future work: to improve the speed of smart grid topology identification and line loss rate calculation. Further efforts will be made with a particular focus on the signal processing capability of edge computing devices in the smart grid cyber-physical system.

## Figures and Tables

**Table 1:** Application of CORDIC Algorithm.

Application Category	Functions Implemented
basic arithmetic	multiplication
	division
trigonometric function	sin *x*
	cos *x*
	tan *x*
inverse trigonometric function	arcsin *x*
	arcsin^−1^ *x*
	arctan^−1^ *x*
hyperbolic function	cosh *x*
	sinh *x*
	tanh *x*
	tanh^−1^ *x*
other common functions	$\sqrt{x}$
	In(*x*)
	e*^x^*
other applications	Fast Fourier transform
	Matrix eigenvalue estimation
	Singular value decomposition
	Digital frequency synthesis

**Table 2:** Features of related CORDIC algorithms.

CORDIC Algorithm	Radix	Rotation Direction Prediction	Fixed Scaling Factor
R-2 CORDIC [26]	R-2	**×**	**√**
R-4 CORDIC [24]	R-4	**×**	**×**
R-8 CORDIC [28]	R-8	**×**	**×**
scaling-free CORDIC [31]	MIX-R	**×**	**√**
Mixed-R-scaling-free CORDIC [29]	MIX-R	**×**	**√**
BBR-CORDIC [34]	R-2	**√**	**×**
CORDIC II [38]	R-2	**×**	**×**
RDP-CORDIC [proposed]	R-2	**√**	**√**

**Table 3:** Prediction constant table for 16-bit precision direction.

{*d*_1_,*d*_2_,*d*_3_,*d*_4_,*d*_5_}	*λ*	*θ*_cp5_
01111	0.03635239	−0.0305780
10000	0.03636256	0.03190169
10001	0.03643358	0.09425964
10010	0.03644375	0.15673931
10011	0.03699740	0.21813201
10100	0.03700756	0.28061168
10101	0.03707859	0.34296963
10110	0.03708875	0.40544930
10111	0.04137373	0.45937935
11000	0.04138389	0.52185901
11001	0.04145492	0.58421697
11010	0.04146508	0.64669663
11011	0.04201873	0.70808934
11100	0.04202890	0.77056900

**Table 4:** Value of *μ_i_* constants for the first 10 iterations.

*i*	*μ_i_*
1	0.0363523910
2	0.0050213369
3	0.0006450055
4	8.1190004043 × 10^−5^
5	1.0166569732 × 10^−5^
6	1.2713795232 × 10^−6^
7	1.5893989889 × 10^−7^
8	1.9868033028 × 10^−8^
9	2.4835211812 × 10^−9^
10	3.1044068054 × 10^−10^

**Table 5:** Angle interval conversion.

Input Angle Range *θ*_*e*_	Angle after Conversion *θ*	cos *θ_e_*	sin *θ_e_*
[0, π/4)	*θ*_e_	cos *θ*	sin *θ*
[π/4, π/2)	π/2 − *θ*_*e*_	sin *θ*	cos *θ*
[π/2,3π/4)	*θ*_*e*_ − π/2	−sin *θ*	cos *θ*
[3π/4, π)	π − *θ*_*e*_	−cos *θ*	sin *θ*
[π,5π/4)	*θ*_*e*_ − π	−cos *θ*	−sin *θ*
[5π/4, 3π/2)	3π/2 − *θ*_*e*_	−sin *θ*	−cos *θ*
[3π/2, 7π/4)	*θ*_*e*_ − 3π/2	sin *θ*	−cos *θ*
[7π/4, 2π]	2π − *θ*_*e*_	cos *θ*	−sin *θ*

**Table 6:** Performance comparison results of different CORDIC algorithms.

CORDIC Algorithm	Number of Iterations	LUTs + FF	ROM	Power (mW)
R-2 CORDIC [26]	17	2362	0	71
R-4 CORDIC [24]	9	1886	24 × 16	66
R-8 CORDIC [28]	7	1566	36 × 16	65
Mixed-R-scaling-freeCORDIC [29]	8	1771	12 × 16	53
BBR-CORDIC [34]	5	1643	102 × 16	82
CORDIC II [38]	7	1433	24 × 16	32
RDP-CORDIC [proposed]	5	1438	12 × 16	28

**Table 7:** Time consumed for the first calculation of the sine and cosine function.

CORDIC Algorithm	Time (ns)
R-2 CORDIC [26]	190
R-4 CORDIC [24]	100
R-8 CORDIC [28]	80
BBR-CORDIC [34]	60
CORDIC II [38]	90
RDP-CORDIC [proposed]	60
